# Supplemental Microalgal DHA and Astaxanthin Affect Astaxanthin Metabolism and Redox Status of Juvenile Rainbow Trout

**DOI:** 10.3390/antiox10010016

**Published:** 2020-12-27

**Authors:** Kun Wu, Beth M. Cleveland, Mark Portman, Wendy M. Sealey, Xin Gen Lei

**Affiliations:** 1 Department of Animal Science, Cornell University, Ithaca, NY 14853, USA; kw393@cornell.edu; 2 USDA/ARS National Center for Cool and Cold Water Research, Leetown, WV 25430, USA; 3 US Fish and Wildlife Service Bozeman Fish Technology Center, Bozeman, MT 59715, USA; mportman21@gmail.com

**Keywords:** algae, astaxanthin, n-3 fatty acid, redox status, trout

## Abstract

Microalgal docosahexaenoic acid (DHA) and astaxanthin (AST) may substitute for fish oil and synthetic AST in aquafeeds. This study explored the effects and mechanisms of those substitutions on AST metabolism and redox status of rainbow trout fed plant protein meal (PM)- or fishmeal (FM)-based diets. Two parallel experiments (PM vs. FM) were performed with 612 juvenile rainbow trout for 16 weeks as a 2 × 3 factorial arrangement of treatments with two AST sources (synthetic (SA) vs. microalgal (AA), at 80 mg/kg) and three levels (0, 50, and 100%) of fish oil substitutions with DHA-rich microalgae. The fish oil substitutions exhibit main effects (*p* < 0.05) and/or interactive effects (*p* < 0.05) with the source of AST on AST deposition, malondialdehyde and glutathione concentrations, and mRNA levels and activities of major redox enzymes (glutathione reductase (GR), glutathione peroxidase (GPX), glutathione S-transferase (GST), and superoxide dismutase (SOD)) in the muscle and liver of trout fed both diet series. The AST source produced only differences in tissue AST deposition (*p* < 0.05) and number of metabolites. In conclusion, the substitutions of fish oil by the DHA-rich microalgae exerted more impacts than those of SA by AA on redox status and functional expression of antioxidant enzymes in the tissues of rainbow trout.

## 1. Introduction

Over the last decade, rapidly growing fish production has led the average consumption of fish to an annualized increase rate of 8% and this is likely to reach 22 kg per capita/year in 2025 [1]. Rainbow trout (*Oncorhynchus mykiss*) is one of the most widely cultured fish species around the world. Over 576 thousand tons of rainbow trout, valued at 2.4 billion dollars, are cultured in 69 countries [2]. Raising rainbow trout as such a vital part of the aquaculture industry relies on dietary fish oil (FO) supplementation to provide long-chain polyunsaturated fatty acid (LC-PUFA) such as docosahexaenoic acid (DHA) and eicosapentaenoic acid (EPA) for ensuring the healthy growth and nutritional value of fish [3]. Shortfalls in supplies and increases in prices of FO have limited the expansion of aquaculture [4]. Meanwhile, astaxanthin (AST) is a red-orange xanthophyll carotenoid abundantly distributed in microalgae and is widely used in the food and feed industry as a pigment [5]. Currently, the most widely (over 95%) used AST sources in animal feeds are synthesized chemically [6]. However, chemical sources of AST may have safety concerns [5,7,8,9] and are susceptible to degradation during storage without being embedded in a matrix with antioxidants [10].

Microalgae are rich sources of natural AST [11] and n-3 LC-PUFA [12]. There have been strong interests in using microalgae to replace FO (DHA) and synthetic AST in feeds. Indeed, microalgal oil was successfully used to replace FO in diets for rainbow trout, resulting in similar growth and tissue DHA deposition [13] but decreased flesh contaminant levels [14]. Our concurrent study demonstrated that the DHA-rich microalgal meal could replace up to 50% FO in diets containing synthetic AST without negative effects on growth performance and tissue fatty acid profiles of juvenile rainbow trout [15]. Microalgal AST (AA) is viewed as a suitable substitution of synthetic AST (SA) because of its excellent pigmentation efficacy and potent antioxidant capacity [7]. Sommer et al. [16] pointed out that SA led to higher AST levels in rainbow trout, whereas others demonstrated that *Haematococcus* microalgae had equal or superior pigmentation compared with SA [17]. However, past studies have mainly investigated singular effects of these substitutions on growth performance, flesh pigmentation, and physiological status of fish [17,18]. To the best of our knowledge, there is no information on the mutual effects or interactions of these substitutions on AST enrichment and metabolism in rainbow trout.

Antioxidant defenses in fish depend upon the supply and coordination of intrinsic and extrinsic antioxidants to scavenge free radicals such as reactive oxygen species (ROS) [19]. Whereas AST is a superior antioxidant that surpasses β-carotene, lutein, and even α-tocopherol [20], DHA can be a strong pro-oxidant due to its exceptionally high unsaturation and oxidative potential in the chemical structure [21]. It remains largely unclear, in particular in fish, as to how sources and (or) concentrations of dietary DHA and AST affect the redox status and antioxidant defenses in fish. Intrinsic or endogenous antioxidant defenses include glutathione (GSH), catalase (CAT), superoxide dismutase (SOD), glutathione peroxidase (GPX), glutathione reductase (GR), and glutathione *S*-transferase (GST).

Therefore, the present study was conducted to evaluate how supplemental microalgal DHA and AST as substitutions of FO and SA affect AST metabolism and enrichment, redox status, and mRNA levels and activities of major antioxidant enzymes in the tissues of juvenile rainbow trout fed plant protein meal (PM)- or fishmeal (FM)-based diets.

## 2. Materials and Methods

### 2.1. Experimental Design and Diet Preparation

The detailed experimental design and diet formulations were presented in a concurrent study paper [15]. Briefly, two parallel 16-week feeding trials were run with a total of 612 juvenile rainbow trout assigned to 36 tanks (3 tanks/treatment by diet, 17 fish/tank, initial body weight = 22 ± 0.26 g). In both PM and FM diet series, a 2 × 3 factorial arrangement of dietary treatments was employed with 2 AST sources (at 80 mg/kg, synthetic, or SA, vs. microalgal, or AA) and three levels (0, 50, and 100%) of fish oil substitution with DHA-rich *Aurantiochytrium* microalgal meal (Heliae, Gilbert, AZ, USA) [19]. SA (4-ascorbyl polyphosphate Rovomix Stay-C 35; Carophyll pink) was purchased from DSM Nutritional Products Ltd. (Basel, Switzerland) and AA was from *Haematococcus pluvialis* (Heliae, Gilbert, AZ, USA) [19]. Fish were handled and treated in accordance with guidelines approved by the Cornell University Animal Care and Use Committee and the U.S. Fish and Wildlife Service (Protocol: Developing a new feed protein complex to replace fishmeal in fish farming, #2017-0072, approved on 10-24-2017).

### 2.2. Fish Culture and Sampling

Rainbow trout from a single lot were obtained from a commercial producer (Troutlodge, Inc., Sumner, WA, USA), and cultured at the Bozeman Fish Technology Center (Bozeman, MT, USA). Lighting was maintained on a 13:11 h diurnal cycle. Fish were acclimated to tanks for 1 week prior to the beginning of the feeding trial. Diets were randomly assigned to three tanks (32, 200 L) per treatment. Tanks were configured in a partial recirculating system with biofiltration, solids removal, and UV treatment of the water. Approximately 25% makeup water was added to the system daily, and water temperature was maintained at 14 °C. Fish were fed to apparent satiation twice a day for 6 days a week for 16 weeks, and feed intake was determined by weighing buckets before and after feeding. Apparent satiation was defined as all the feed the fish consumed in a 30-min period. At the end of the study, liver and muscle samples were collected, frozen in liquid nitrogen, and then stored at −80 °C for the subsequent biochemical and molecular analyses.

### 2.3. Astaxanthin Analysis

Total AST in diet and tissues was extracted using a published protocol [22] with modifications. Briefly, samples of tissues (0.5–2 g) and diet (0.5–1 g) were homogenized in ethyl acetate, followed by incubation for 10 min on ice with vortexing periodically. Water was then added and centrifuged at 3000× *g* for 15 min at 4 °C. The upper phase containing AST was collected and dried under nitrogen gas. All residues were dissolved in chloroform (HPLC-grade) and then filtered for HPLC-UV analysis. The AST concentrations were measured according to methods of Sowell et al. [23], Breithaupt et al. [24], and Rohrle et al. [25] with modifications. The AST extract was eluted with methanol and acetonitrile (50:50) containing 0.1% triethylamine (TEA) at a flow rate of 1 mL/minute on an Agilent Eclipse plus C18 reverse-phase column (5 μm, 4.6 × 250 mm, at 30 °C) using an Agilent HPLC system with an LC-10AD micro plunger pump and an SPD-10 AV vp UV detector. The eluted peaks were identified by comparison of the retention times of standard AST (Sigma, St. Louis, MO, USA). To validate the results, sample extracts were spiked with standard AST to determine its appearance on the chromatogram in relation to the sample peak being identified. The AST concentrations were calculated by the areas under the curves (AUCs) of the samples against those of the spiked AST standard in the chromatograms. The extraction efficiency (recovery) of AST was 92%.

### 2.4. Determination of Antioxidant/Oxidant Biomarkers

Malondialdehyde (MDA) determination was based on the reaction of thiobarbituric acid (TBA) with MDA and the formation of fluorescing (excitation/emission 520/550 nm) MDA-(TBA)_2_ adducts. Glutathione determination was based on the reaction of GSH with DTNB [5,5-dithiobis(2-nitrobenzoic acid)] to form GSSG (glutathione disulfide) and 5-thionitrobenzoic acid (TNB), which was detected spectrophotometrically at 412 nm [19,26]. Activities of GST, GPX, GR, and SOD were measured in the muscle (fillet) and liver tissues using methods described in previous studies [27,28,29,30]. Briefly, GST activity was determined by measuring the formation of the conjugate of GSH and 1-chloro-2,4-dinitrobenzene (CDNB). GPX activity was determined spectrophotometrically at 340 nm by coupling the recycling of GSH with the oxidation of NADPH under the catalysis of GR. GR activity was determined by measuring the rate of NADPH oxidation used for reducing GSSG at 340 nm. SOD activity was measured by a spectrophotometric method (470 nm) based on the inhibition of XTT (3′-{1-[(Phenylamino)-carbonyl]-3,4-tetrazolium}-bis(4-methoxy-6-nitro)benzenesulphonic acid hydrate) reduction by superoxide anions generated by xanthine-xanthine oxidase.

### 2.5. Gene Expression

Total RNA of the liver was extracted using TRIzol reagent (Millipore-Sigma, St. Louis, MO, USA) according to the manufacturer’s protocol. Quantitative real-time PCR (qPCR) was performed using an ABI Vii 7 Detection System (Applied Biosystems, Foster City, CA, USA) using SYBR Green supermix (Bio-Rad, Hercules, CA, USA). The reaction volume was 20 μL, which contained 1 μL 10-fold dilution of cDNA (complementary DNA), 10 μL SYBR Green supermix, 10 mM each of forward and reverse primers (Table S1), and 0.4 and 8.2 µL H_2_O. The qPCR protocol consisted of initial denaturation at 95 °C for 30 s, followed by 40 cycles at 95 °C for 5 s, and 60 °C for 60 s. Real-time qPCR data were analyzed using the standard curve approach after normalizing to beta-actin and *elf1a* (elongation factor 1a) as reference genes.

### 2.6. Statistical Analysis

Because of the main objectives of the present study and the ingredient differences and the resultant impact on the responses to treatments, we analyzed the data from the PM and FM diet series separately. Within each diet series, a two-way ANOVA (analysis of variance) (2 by 3 factorial arrangement of dietary treatments) was used to evaluate the main effects of AST source (SA vs. AA), fish oil substitution levels (0, 50, and 100%), and their interactions. Duncan’s multiple range test was used to compare the treatment mean differences. Pearson’s correlation was calculated to examine the relationship between AST concentration and MDA, GSH, and enzyme activities within tissues. Three types of stepwise regression analyses were performed to rank the importance of the assumed independent variables such as tissue AST concentration in regulating the responses of assumed dependent variables such as tissue MDA or GSH or activity of redox enzymes. Data are presented as means ± SEM (standard error of mean), and the significance level is *p* < 0.05.

## 3. Results

Detailed composition and analytic nutrient values including DHA concentrations of experimental diets were reported in the concurrent study paper [15]. Effects of the FO and SA substitutions on growth performance, body condition indices, tissue lipid and fatty acid profiles, and gene expression related to growth and biosynthesis of LC-PUFA were presented in that paper. Notably, the 100% FO oil replacement impaired growth performance, dietary protein and energy utilization, body indices, and tissue accumulation of DHA and EPA in both diet series. Overall, replacing FO with DHA-rich microalgae produced more negative metabolic responses than the substitution of SA by AA in fish fed both PM and FM diet series.

### 3.1. Concentrations of Astaxanthin in Diets and Tissues

**Figure 1:**
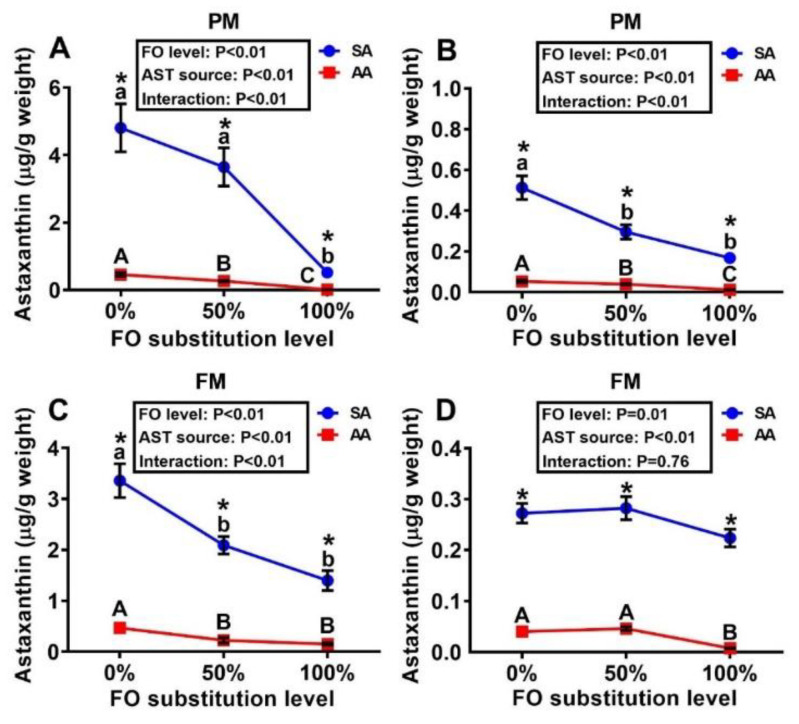
Effects of dietary fish oil (FO) substitution by docosahexaenoic acid (DHA)-rich microalgae and source of astaxanthin (AST) on astaxanthin concentrations in the muscle (fillet, **A**,**C**) and liver (**B**,**D**) of juvenile rainbow trout fed the plant meal- (PM, **A**,**B**) and fishmeal (FM, **C**,**D**)-based diets. Means labelled with different letters [lowercase (a, b), synthetic astaxanthin (SA) groups; uppercase (A–C), microalgae astaxanthin (AA) groups] are different between various fish oil substitutions (*p* < 0.05). Asterisks (*) indicate differences between SA and AA groups (*p* < 0.05) at the same level of FO substitution. FO: fish oil. AST: astaxanthin.

In both the PM and FM diet series, the SA diets had > five-fold higher (*p* < 0.05) concentrations of AST than those of AA (Table 1). Consequently, similar differences (*p* < 0.05) between SA and AA diets were shown in both liver and muscle accumulations of AST (Figure 1). Within a given source of AST, there was a progressive reduction (*p* < 0.05) in liver and muscle AST concentrations with increasing levels of FO substitution with the DHA-rich microalgae, although the magnitudes of the decreases varied with the treatment combination and tissue type. Fish fed the PM diets supplemented with SA and 0% FO substitution had the highest AST accumulations, reaching 4.8 μg/g of the muscle and 0.5 μg/g of the liver.

### 3.2. Metabolites of Astaxanthin in the Liver and Muscle

**Figure 2:**
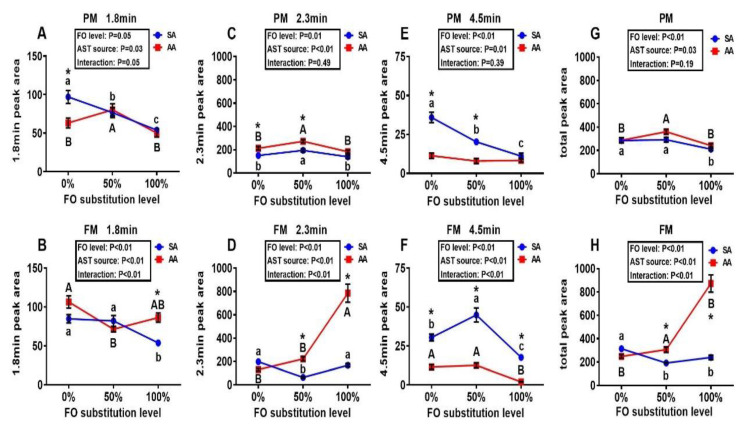
Effects of dietary fish oil substitution by DHA-rich microalgae and source of astaxanthin on relative areas of hepatic astaxanthin metabolite peaks (**A**,**B**: 1.8 min; **C**,**D**: 2.3 min; **E**,**F**: 4.5 min; **G**,**H**: total of the three peaks) in juvenile rainbow trout fed the plant meal- (PM, **A**,**C**,**E**,**G**) and fishmeal (FM, **B**,**D**,**F**,**H**)-based diets. Means labelled withdifferent letters [lowercase (a, b), synthetic astaxanthin (SA) groups; uppercase (A, B), microalgae astaxanthin (AA) groups] are different between various levels of fish oil substitution (*p* < 0.05). Asterisks (*) indicate differences between SA and AA groups (*p* < 0.05) at the same level of FO substitution. The absence of different letters (a, b, c or A, B) or asterisks (*) between means indicates no significant differences. FO: fish oil. AST: astaxanthin.

Only a single peak of AST (retention time: 3.8 min) was found in the HPLC chromatogram profiles of muscle samples from all treatment groups. In comparison, three peaks (retention times: 1.8, 2.3, and 4.5 min), in addition to the same peak shown in the muscle, were observed in the liver samples (Figure 2). These peaks presumably represented different metabolites of AST. The 1.8 min peak area was decreased (*p* < 0.05) by the 100% FO substitution in the SA diets of both PM and FM series (Figure 2A,B). The AA diets resulted in greater (*p* < 0.05) 2.3 min peak areas than those of SA in both PM and FM series (Figure 2C,D). Intriguingly, this peak area was enhanced (*p* < 0.05) in the liver of fish by 100% FO substitution over the 0% or 50% FO substitution in the AA diets of FM series (Figure 2D). The SA diets led to greater (*p* < 0.05) areas of the 4.5 min peak than the AA diets in both PM and FM series, and the 100% FO substitution decreased (*p* < 0.05) the area of this peak compared with the other two levels of FO substitution (Figure 2E,F). Responses of the total areas of the three peaks to the treatments seemed to be similar to those of the 2.3 min peak (Figure 2G,H). Overall, both the AST source and the FO substitution exhibited main effects (*p* < 0.01), but no interactions (except for the 1.8 min peak), on the areas of these peaks either singularly or together in the PM series. In contrast, the AST source and the FO substitution demonstrated not only main effects (*p* < 0.01) but also interactions (*p* < 0.01) on the areas of all these peaks in the FM series.

### 3.3. Tissue MDA and GSH Concentrations and Antioxidant Enzyme Activities

**Figure 3:**
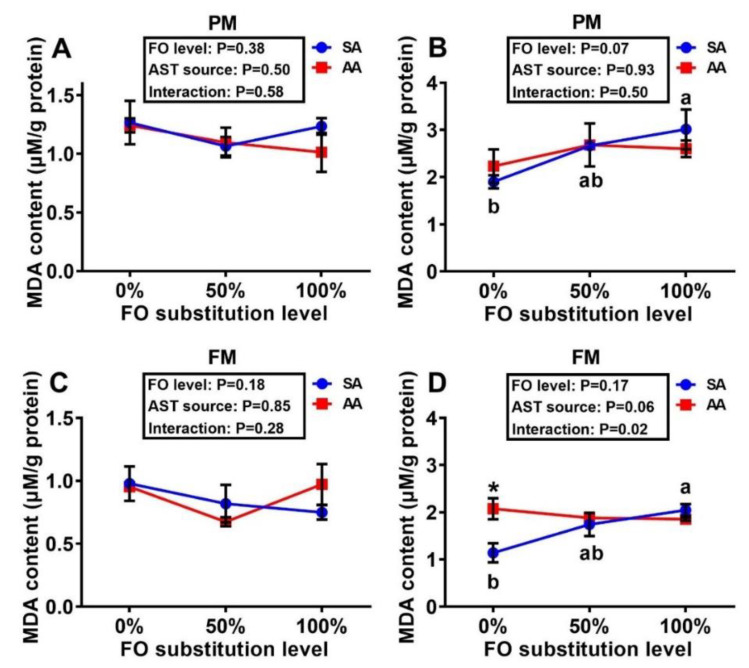
Effects of dietary fish oil substitution by DHA-rich microalgae and source of astaxanthin on MDA concentrations in the muscle (fillet, **A**,**C**) and liver (**B**,**D**) of juvenile rainbow trout fed the plant meal- (PM, **A**,**B**) and fishmeal (FM, **C**,**D**)-based diets. Means labelled with different letters [lowercase (a, b), synthetic astaxanthin (SA) groups] are different between various fish oil substitutions (*p* < 0.05). Asterisks (*) indicate differences between the SA and AA groups (*p* < 0.05) at the same level of FO substitution. The absence of different letters (a, b) or asterisks (*) between means indicates no significant differences. MDA: malondialdehyde. FO: fish oil. AST: astaxanthin.

**Figure 4:**
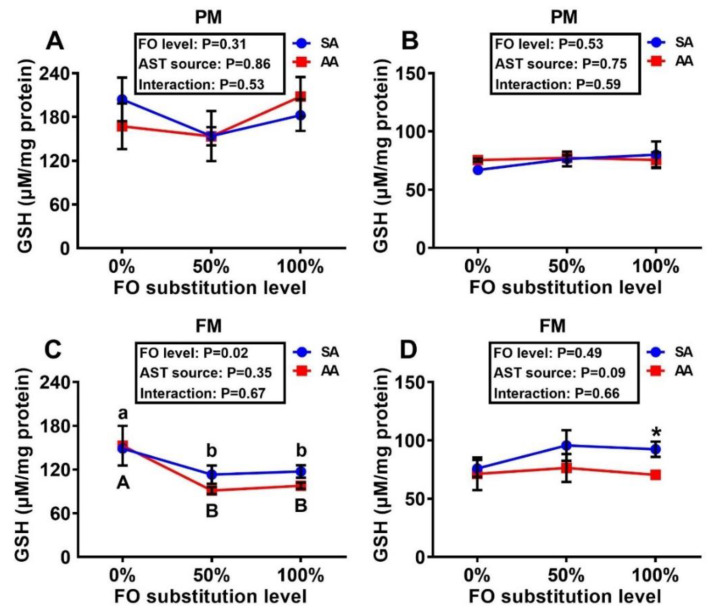
Effects of dietary fish oil substitution by DHA-rich microalgae and source of astaxanthin on GSH concentrations in the muscle (fillet, **A**,**C**) and liver (**B**,**D**) of juvenile rainbow trout fed the plant meal- (PM, **A**,**B**) and fishmeal (FM, **C**,**D**)-based diets. Means labelled with different letters [lowercase (a, b), synthetic astaxanthin (SA) groups; uppercase (A, B), microalgae astaxanthin (AA) groups] are different between various fish oil substitutions (*p* < 0.05). Asterisks (*) indicate differences between the SA and AA groups (*p* < 0.05) at the same level of FO substitution. The absence of different letters (a, b or A, B) or asterisks (*) between means indicates no significant differences. GSH: glutathione. FO: fish oil. AST: astaxanthin.

There were dose-dependent elevations in the liver MDA concentrations with increasing levels of FO substitution in the fish fed the SA diets of both PM and FM series (Figure 3B,D), whereas the muscle MDA concentrations remained similar across the dietary treatments (Figure 3A,C). Muscle GSH concentrations in the fish fed the FM diet series were decreased (*p* < 0.05) by the 50% and 100% substitutions of FO over the 0% substitution (Figure 4C). In fish fed the PM diet series, the FO substitutions resulted in dose-dependent activity decreases (*p* < 0.05) of all four assayed enzymes in both muscle and liver, except for muscle GPX and liver GR activities (Table 2). In contrast, the AST source exerted a main effect (*p* < 0.05) on only liver GST activity, in which the SA supplementation led to lower (*p* < 0.05) hepatic GST activities than that with AA at both 0% and 50% FO substitutions. In fish fed the FM diet series, activities of the four assayed enzymes were not altered in the muscle by the dietary treatments. In contrast, hepatic activities of these enzymes were affected (*p* < 0.05) by the FO substitutions, the AST source, and (or) their interactions. In general, 50% and (or) 100% FO substitution decreased (*p* < 0.05) the activities more than the 0% substitution, whereas SA produced higher (*p* < 0.05) activity levels than AA with a couple of exceptions.

### 3.4. Antioxidant Enzyme Gene Expressions

**Figure 5:**
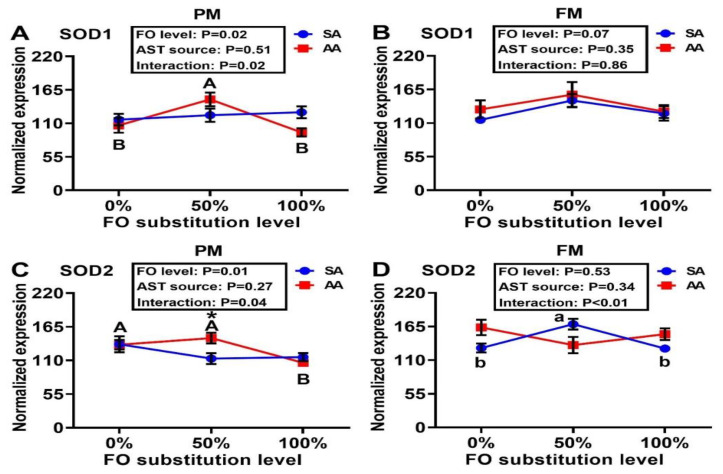
Effects of dietary fish oil substitution by DHA-rich microalgae and source of astaxanthin on hepatic mRNA levels of SOD1 (**A**,**B**) and SOD2 (**C**,**D**) of juvenile rainbow trout fed the plant meal- (PM, **A**,**C**) and fishmeal (FM, **B**,**D**)-based diets. Means labelled with different letters [lowercase (a, b), synthetic astaxanthin (SA) groups; uppercase (A, B), microalgae astaxanthin (AA) groups] are different between various fish oil substitutions (*p* < 0.05). Asterisks (*) indicate differences between SA and AA groups (*p* < 0.05) at the same level of FO substitution. The absence of different letters (a, b or A, B) or asterisks (*) between means indicates no significant differences. SOD: superoxide dismutase. FO: fish oil. AST: astaxanthin.

**Figure 6:**
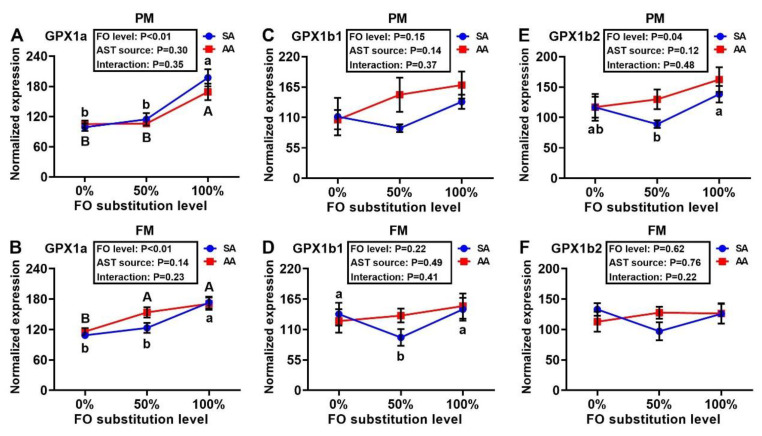
Effects of dietary fish oil substitution by DHA-rich microalgae and source of astaxanthin on hepatic mRNA levels of GPX1a (**A**,**B**), GPX1b1 (**C**,**D**), and GPX1b2 (**E**,**F**) of juvenile rainbow trout fed the plant meal- (PM, **A**,**C**,**E**) and fishmeal (FM, **B**,**D**,**F**)-based diets. Means labelled with different letters [lowercase (a, b), synthetic astaxanthin (SA) groups; uppercase (A, B), microalgae astaxanthin (AA) groups] are different between various fish oil substitutions (*p* < 0.05). The absence of different letters (a, b or A, B) between means indicates no significant differences. GPX: glutathione peroxidase. FO: fish oil. AST: astaxanthin.

**Figure 7:**
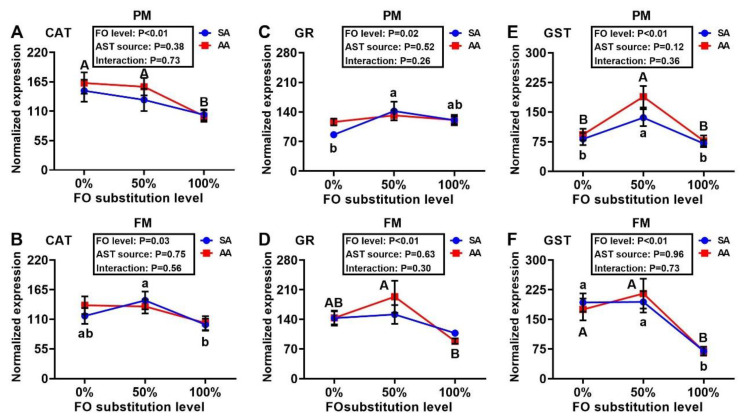
Effects of dietary fish oil substitution by DHA-rich microalgae and source of astaxanthin on hepatic mRNA levels of CAT (**A**,**B**), GR (**C**,**D**), and GST (**E**,**F**) of juvenile rainbow trout fed the plant meal- (PM, **A**,**C**,**E**) and fishmeal (FM, **B**,**D**,**F**)-based diets. Means labelled with different letters [lowercase (a, b), synthetic astaxanthin (SA) groups; uppercase (A, B), microalgae astaxanthin (AA) groups] are different between various fish oil substitutions (*p* < 0.05). The absence of different letters (a, b or A, B) between means indicates no significant differences. CAT: catalase; GR: glutathione reductase; GST: glutathione S-transferase. FO: fish oil. AST: astaxanthin.

Expression of SOD1 was similar across treatment groups, with the exception of the PM–AA diet series in which the 50% FO substitution led to greater (*p* < 0.05) SOD1 expression than the 0% or 100% FO substitutions (Figure 5A,B). The 100% FO substitution decreased (*p* < 0.05) hepatic expression of SOD2 more than the 0% and 50% FO substitutions in the PM–AA diet series. At 50% FO substitution, AA produced greater (*p* < 0.05) hepatic mRNA levels of SOD2 than SA. In the FM–SA diet series, the 50% FO substitution induced higher (*p* < 0.05) expression of SOD2 than that of 0% or 100% substitution (Figure 5C,D). In both the FM and PM diet series, the FO substitutions produced dose-dependent positive effects on the expression of hepatic GPX1a regardless of the AST source (Figure 6A,B). Neither the FO substitution nor the AST source had main effects on the expressions of liver GPX1b1 (Figure 6C,D), although a main effect of FO substitution on hepatic GPX1b2 expression occurred in the PM diet series (Figure 6E,F). Main effects (*p* < 0.05) of FO substitution occurred on hepatic CAT expression in both the PM and FM diet series, in which lower (*p* < 0.05) expression levels were shown in the 100% than the 0% and 50% FO substitution groups of the PM–AA and FM–SA series (Figure 7A,B). Main effects (*p* < 0.05) of the FO substitution also occurred on hepatic GR expression. In the PM-SA diet series, the GR expression was elevated (*p* < 0.05) by the 50% than the 0% FO substitution, whereas the expression was decreased (*p* < 0.05) by the 100% than the 50% FO substitution in the FM-AA diet series (Figure 7C,D). In the PM diet series, hepatic GST expression was greater (*p* < 0.05) with 50% rather than 0% or 100% FO substitution, regardless of the AST source (Figure 7E,F). In the FM diet series, hepatic GST expression was much lower (*p* < 0.0001) with 100% rather than 0% or 50% FO substitution, irrespective of the AST source. Notably, the AST source failed to show main effects on the hepatic expression of any of the eight genes assayed.

### 3.5. Correlation Analyses

In the PM–AA diet series, there were strong positive correlations (R > 0.74, *p* < 0.05) between the AST concentrations and activities of GST, GR, and SOD in the muscle (Table S2). In the FM–SA diet series, there was a strong postivie correlation (*R* = 0.70, *p* < 0.05) between the muscle AST and GSH concentrations. Liver AST concentrations were negatively correlated with the MDA concentrations (*R* = −0.83, *p* < 0.05), but positively associated with SOD activity (*R* = 0.82, *p* < 0.05) in the PM–SA diet series. There was a positive correlation (*R* = 0.87, *p* < 0.05) between the liver AST concentrations and GST activities in the PM–AA diet series. There were positive correlations between the liver AST concentrations and GPX (*R* = 0.67, *p* < 0.05) and SOD (*R* = 0.90, *p* < 0.05) activities in the FM–AA series.

### 3.6. Stepwise Regression Analyses

In the first analysis of stepwise regression, the analyzed dietary concentrations of DHA [15] and AST were used as independent variables and the tissue concentrations of MDA and GSH and activities of GST, GPX, GR, and SOD were used as dependent variables (Table S3). The results indicated that the dietary DHA concentration was the remaining significant variable that positively affected activities of muscle GST (*R* = 0.74, *p* < 0.05) and SOD (*R* = 0.91, *p* < 0.05) and liver SOD (*R* = 0.82, *p* < 0.05) in the PM diet series. Similarly, only the dietary DHA but not AST concentration showed a positive association with activities of muscle GST (*R* = 0.84, *p* < 0.05) and GR (*R* = 0.74, *p* < 0.05) and liver GPX (*R* = 0.78, *p* < 0.05) in the FM diet series.

In the second analysis, the dependent variables were the same as in the first analysis whereas the independent variables included the tissue concentrations of DHA [15] and AST and liver mRNA levels of the eight antioxidant enzyme genes (Table S4). In the PM diet series, the muscle MDA concentration was negatively correlated with hepative GR mRNA level (*R* = 0.74, *p* < 0.05), and the muscle GST (*R* = 0.84, *p* < 0.05), GR (*R* = 0.83, *p* < 0.05), and SOD (*R* = 0.96, *p* < 0.05) activities were positively affected by hepatic mRNA levels of catalase. In comparison, liver MDA concentrations and activities of GR and SOD were positively affected (*R* > 0.77, *p* < 0.05) by liver concentrations of GST or DHA [15]. Liver GSH concentrations and GPX and GST activities were positively affected (*R* > 0.75, *p* < 0.05) by mRNA levels of three different antioxidant enzyme genes. In the FM diet series, muscle GSH concentrations and GST, GR, and SOD activities were affected (*R* > 0.75, *p* < 0.05) by hepatic mRNA levels of three antioxidant enzyme genes respectively or jointly. Liver GSH concentrations (*R* = 0.75, *p* < 0.05) and GR activities (*R* = 0.97, *p* < 0.05) were affected by liver concentrations of AST and DHA [15], respectively. Liver activites of the four antioxidant enzymes were affected (*R* > 0.90, *p* < 0.05) by hepatic mRNA levels of four different antioxidant enzyme genes.

## 4. Discussion

It is novel to show the main effects of FO substitution by DHA-rich microalgae and its interaction effects with the source of AST on tissue MDA and GSH concentrations and activities of GST, GPX, GR, and SOD. There were dose-dependent elevations of MDA in the liver of trout fed the SA diets with increasing levels of FO substitutions. As MDA is an end-product of lipid peroxidation, the rise in MDA implies accelerated oxidative stress [31]. In comparison, trout fed the AA diets maintained similar liver MDA concentrations across different levels of FO substitution, despite higher baseline values than those fed the SA diet at the 0% FO substitution. Likewise, the FO substitution largely suppressed activities of the assayed antioxidant enzymes in liver and (or) muscle with a few exceptions. The suppression was relatively stronger or more consistent with the GST and SOD rather than GPX and GR activities in either PM or FM diet series. The stepwise regression analyses also reinforced the importance of dietary DHA concentrations [15] (due to the FO substitution by microalgal DHA) in upregulating tissue activities of antioxidant enzymes. Previous studies also indicated that DHA enhanced antioxidative enzyme activities [32,33]. Structurally, DHA has a number of double bonds that are susceptible to oxidation [34]. Compared with the 50% and 100% FO substitutions, the 0% FO substitution diet contained a higher concentration of DHA [15] that might induce more oxidation reactions, resulting in upregulation of antioxidant enzymes to maintain the redox balance [35]. In contrast, the AST source showed little or no consistent effects on tissue GSH concentrations and antioxidant enzyme activities. This outcome was somewhat different from our expectations that intrinsic antioxidant defense [19] in tissues might be altered by or respond to supplemental SA and AA differently. In fact, supplemental AST was shown to enhance body antioxidant capacity of aquatic species [36,37,38] and mammals [39,40,41] in a number of studies, although the opposite was reported in other studies [19,42,43,44] as a coordination between the intrinsic and extrinsic antioxidants [43]. However, there were strong positive correlations between the AST concentrations and the antioxidant enzyme activities and GSH concentrations in the muscle and (or) liver, despite variations with the diet treatments. This implies that once AST was absorbed from the diet and transported to tissues, it could exhibit greater antioxidant or redox-modulating potential. Because FO substitution downregulated the tissue AST deposition, part of its inhibition of antioxidant enzyme activities could be attributed to the resultant AST declines in the tissues. Therefore, the impacts of dietary AST source or concentration on tissue antioxidant enzyme activities and redox balance are not unilateral but multi-lateral.

Dietary AST source produced no significant differences in the mRNA abundances of the assayed antioxidant enzyme genes. Because the AST concentrations in the AA diets were lower than those of the SA diets, AA was presumably more functionally potent than SA on the same mass basis in regulating these genes. A previous study suggested that SA needed to be used at a rate 14–55 times greater than AA to obtain the equivalent antioxidant protection [7]. Likewise, FO substitution markedly decreased mRNA levels of SOD2 and CAT in the PM diet series and mRNA levels of GR and GST in the FM diet series. This could be partially attributed to declined AST concentrations by FO substitution, as AST was found to upregulate the expression of tissue antioxidant protein genes [36,37,45]. Yang et al. [46] suggested that AST could improve nuclear factor E2-related factor 2 (Nrf2)-mediated endogenous antioxidant defense. Activation of the Nrf2 pathway enhanced GSH concentrations and gene expression of antioxidant enzymes such as GPX, GST, and GR [47,48]. Plausibly, Nrf2 might constitute a link between the tissue AST deposition and antioxidant enzyme responses in the present study. However, further studies are needed to investigate whether and how AST activates the Nrf2 pathway in trout. Intriguingly, in both FM and PM diet series, the 100% FO substitution upregulated GPX1a expression compared with the 0% FO substitution, which was opposite to the GPX enzyme activity changes. It is plausible that upregulated mRNA abundance represented a compensatory response to the depressed GPX activity, which helped maintain the redox homeostasis. The stepwise regression showed that GPX1b1 and GPX1b2 were positively correlated with GPX and GST activities, whereas the GPX1a mRNA was rendered as the negative variable for activities of several antioxidant enzymes. These results reflect different responses and sensitivities of various GPX gene isoforms [49]. Similar effects of GPX1b1 and GPX1b2 may be attributed to their closer relationship of gene sequences compared with GPX1a [50]. Furthermore, the stepwise regression analyses indicated that the hepatic CAT and GR mRNA levels might be relevant biomarkers to reflect muscle antioxidant status in the PM diet series. This notion was supported by a negative correlation between the liver GR expression and muscle MDA concentrations and a positive correlation between liver CAT expression and muscle activities of GST, GR, and SOD. Notably, the muscle and liver AST and DHA concentrations [15] showed different correlations with antioxidant biomarkers from those with dietary AST and DHA concentrations. Dietary AST concentrations showed no association with GSH concentration or antioxidant enzyme activities in the muscle or liver, whereas liver AST concentration was positively correlated with the liver GSH concentration in the FM diet series. Dietary DHA concentration remained as a positive variable of many antioxidative enzymes; however, liver DHA concentration was shown as the positive factor of only hepatic GR activity in the FM diet series. These divergent effects indicate the complexity of regulation of redox status between extrinsic foods and intrinsic biological systems [51]. As for DHA, its oxidative potential varies with environment, such as in micelles, emulsion, cell membrane, or biological systems, and depends on the relative location of the lipid substrate and interaction of antioxidants and pro-oxidants [51]. It seems that the interaction of DHA and AST and the change in environment strengthened the correlations between the antioxidant enzymes and tissue AST concentrations but weakened such correlations with tissue DHA concentrations.

Our results indicate that the FO substitution inhibited the enrichment of AST in the liver and muscle. The inhibition was particularly evident in the muscle of fish fed the PM diet series where the muscle AST concentration in the 100% FO substitution group dropped by as much as 89% (SA-fed) and 96% (AA-fed) compared with the respective 0% substitution groups. Although there was no clear reason to explain the difference in the sensitivities to the FO substitution and concentration changes in the diets between the two tissues, the muscle accumulated nearly ten-fold the amount of AST in the liver on the basis of per gram tissue. Maoka et al. [52] demonstrated that Salmonidae fish accumulated AST in the muscle in a species-specific fashion. Whereas integument tissues and gonads are the primary sites of carotenoid deposition in fish [52], liver seemed to have the highest enrichment of AST among several tissues of broiler chicks [19] and mice [53]. As a fat-soluble xanthophyll carotenoid, AST and lipids are closely related in absorption and metabolism [54]. A high level of lipids or unsaturated fatty acids is favorable to the incorporation of carotenoids [54,55,56]. Chimsung et al. [57] investigated the effects of carotenoids, plant sterols, fiber, cholesterol, and vitamin E on AST absorption in salmon and found that only cholesterol enhanced AST absorption. Seemingly, the lack of cholesterol in the microalgal DHA supplementation, in comparison with the FO [58], might have contributed to the decrease in AST absorption and deposition in the 100% FO substitution groups. In a parallel study [15], we found that FO substitutions with DHA-rich microalgae in both PM and FM diet series decreased concentrations of one or more monounsaturated fatty acids, in agreement with the finding that the fillet concentration of astaxanthin was positively correlated to supplementary oil content of monounsaturated fatty acids in salmon [59]. Furthermore, the absorption and deposition of AST might also be affected by the differences in lipid composition at the different FO substitution levels [60].

Trout fed with SA had higher AST deposition in the liver and muscle than those fed with AA in both diet series. This pattern was similar to that found in other studies [61,62] and might be explained by higher concentrations or portions of free AST in SA than AA that could be accumulated directly in the tissues. In contrast, AA was supposed to contain various carotenoids aside from AST such as β-carotene, canthaxanthin, lutein, and echinenone [54] that require a series of conversions into AST [52]. Microalgal AST was reported to have multiple isomers and complex forms. The Z isomer was less efficiently deposited than the E isomers [5] and the esterified AST was less efficiently absorbed than the unesterified AST [63]. In addition, the thick cell walls of microalgae may impede the digestibility and absorption of the pigments by fish [64]. In line with these chemical form variations, we revealed different metabolic profiles of AA and SA in the liver. Future study is warranted to characterize the chemical structure and metabolic function of those detected new peaks.

## 5. Conclusions

Gene expressions and activities of major antioxidant enzymes were suppressed by FO substitution with DHA-rich microalgae in the liver and muscle of rainbow trout. The FO substitution also decreased the deposition of AST from either SA or AA. Whereas tissue AST concentrations were correlated with the intrinsic antioxidant enzyme activities, dietary source and (or) concentration of AST exerted no major or consistent effect on functional expression of antioxidant enzymes in the liver or muscle of trout. The FO and SA substitutions showed moderate interaction effects on the responses of redox status biomarkers in the tissues of trout, which varied with the major protein type (PM vs. FM) in the diets.

## Figures and Tables

**Table 1:** Analyzed concentrations of astaxanthin in experimental diets ^1^.

	Diets						
Fish Oil Replacement Level ^2^	0%		50%		100%		SEM
Astaxanthin Source ^3^	SA	AA	SA	AA	SA	AA	
Plant meal-based diet							
Astaxanthin (μg/g diet)	42 ^a^	6.7 ^b^	33 ^a^	7.1 ^b^	32 ^a^	4.1 ^b^	0.39
Fishmeal-based diet							
Astaxanthin (μg/g diet)	41 ^a^	5.8 ^b^	37 ^a^	5.1 ^b^	28 ^a^	4.0 ^b^	0.42

^1^ Values of means (*n* = 3); ^2^ Percent of DHA-rich microalgae (*Aurantiochytrium,* Heliae, Gilbert, AZ, USA) to replace menhaden fish oil (Omega Protein, Houma, LA, USA); ^3^ SA: synthetic astaxanthin, 4-ascorbyl polyphosphate Rovomix Stay-C 35; Carophyll pink (DSM Nutritional Products Ltd., Basel, Switzerland); AA: microalgal astaxanthin, *Haematococcus pluvialis*, (Heliae, Gilbert, AZ, USA). Means labelled with different letters (a, b) are different between various diets within the same series (*p* < 0.05).

**Table 2:** Effects of dietary fish oil substitution by DHA-rich microalgae and source of astaxanthin on antioxidant enzyme activities in the muscle (fillet) and liver of juvenile rainbow trout.

	Diets						*p* Value			
Fish Oil Replacement Level ^1^	0%		50%		100%		FO Level	AST Source	Interaction	
Astaxanthin Source ^2^	SA	AA	SA	AA	SA	AA				SEM
**Plant Meal-Based Diet**										
Muscle										
GST ^3^ (mU/mg protein)	0.81 ^a,b^	1.1 ^a^	0.90 ^a^	0.85 ^a,b^	0.72 ^a,b^	0.54 ^b^	0.03	0.90	0.15	0.05
GPX (mU/mg protein)	2.9 ^a,b^	3.4 ^a^	2.1 ^b^	2.9 ^a,b^	2.3 ^a,b^	2.5 ^a,b^	0.15	0.11	0.71	0.16
GR (mU/mg protein)	0.26 ^a,b^	0.32 ^a^	0.19 ^b^	0.24 ^a,b^	0.18 ^b^	0.20 ^b^	0.02	0.13	0.71	0.02
SOD (mU/mg protein)	23 ^a^	24 ^a^	19 ^a,b^	24 ^a^	16 ^a,b^	12 ^b^	0.01	0.92	0.29	1.4
Liver										
GST (mU/mg protein)	4.6 ^a,b,c^	5.7 ^a,b^	4.4 ^b,c^	6.1 ^a^	3.9 ^c^	3.9 ^c^	0.03	0.04	0.24	0.26
GPX (mU/mg protein)	13 ^a,b^	16 ^a^	16 ^a^	14 ^a,b^	11 ^b^	12 ^a,b^	0.04	0.50	0.22	0.62
GR (mU/mg protein)	2.5	2.0	2.7	3.2	2.5	2.6	0.19	0.85	0.38	0.15
SOD (mU/mg protein)	139 ^a,b^	143 ^a^	99.0 ^b,c^	120 ^a,b,c^	88.0 ^c^	105 ^a,b,c^	0.01	0.22	0.80	6.64
**Fishmeal-Based Diet**										
Muscle										
GST (mU/mg protein)	1.0	1.1	0.85	0.84	0.84	0.86	0.03	0.89	0.95	0.04
GPX (mU/mg protein)	2.4	2.4	2.5	1.7	2.8	2.3	0.15	0.11	0.71	0.14
GR (mU/mg protein)	0.26	0.21	0.23	0.18	0.16	0.21	0.49	0.77	0.15	0.01
SOD (mU/mg protein)	26	14	16	21	22	19	0.78	0.25	0.10	1.54
Liver										
GST (mU/mg protein)	5.2 ^a^	4.0 ^a,b^	2.2 ^b^	2.2 ^b^	3.7 ^a,b^	3.1 ^b^	0.01	0.24	0.61	0.31
GPX (mU/mg protein)	14 ^a^	15 ^a^	15 ^a^	9.3 ^b^	9.8 ^b^	5.3 ^c^	<0.001	0.01	0.04	0.98
GR (mU/mg protein)	2.8 ^b,c^	4.0 ^a^	3.6 ^a,b^	2.3 ^c^	2.8 ^b,c^	2.3 ^c^	0.03	0.24	0.01	0.18
SOD (mU/mg protein)	61 ^b,c^	96 ^a^	92 ^a^	86 ^a,b^	56 ^c^	36 ^c^	0.01	0.66	0.02	5.99

^1^ Percent of DHA-rich microalgae (*Aurantiochytrium*, Heliae, Gilbert, AZ, USA) to replace menhaden fish oil (Omega Protein, Houma, LA, USA). ^2^ SA: synthetic astaxanthin, 4-ascorbyl polyphosphate Rovomix Stay-C 35; Carophyll pink (DSM Nutritional Products Ltd., Basel, Switzerland); AA: microalgal astaxanthin, *Haematococcus pluvialis*, (Heliae, Gilbert, AZ, USA). ^3^ GST: Glutathione S-transferase; GPX: Glutathione peroxidase; GR: Glutathione reductase; SOD: Superoxidase dismutase. ^a^, ^b^, ^c^ Values are means (*n* = 3), and means in the same row without a common letter differ (*p* < 0.05).

## Data Availability

Data is contained within the article.

## Supplementary Materials

The following are available online at https://www.mdpi.com/2076-3921/10/1/16/s1, Table S1: Primer sequences for qPCR. Table S2: Correlations between astaxanthin concentrations and MDA and GSH concentrations and antioxidant enzyme activities in the muscle and liver of rainbow trout. Table S3: Stepwise regression analysis of tissue redox biomarkers as dependent variables and dietary AST and DHA concentrations as independent variables in rainbow trout. Table S4: Stepwise regression analysis of tissue redox biomarkers as dependent variables and tissue AST and DHA concentrations and antioxidant enzyme gene mRNA levels as independent variables in rainbow trout.
