# Supplementary material for: Supplemental Microalgal DHA and Astaxanthin Affect Astaxanthin Metabolism and Redox Status of Juvenile Rainbow Trout
Source: Antioxidants (Basel). 2020 Dec 27;10(1):16. doi: 10.3390/antiox10010016 (PMC7823529; doi:10.3390/antiox10010016)
Supplement: Supplementary file 1 [file antioxidants-10-00016-s001.pdf]

Table S1. Gene accession numbers and primer sequences for transcript targets

| Gene | Forward Primer | Reverse Primer | Accession Number | Product length |
|---|---|---|---|---|
| **sod1** | TGGTCCTGTGAAGCTGATTG | TTGTCAGCTCCTGCAGTCAC | AF469663.1 | 201 |
| **sod2** | TCCCTGACCTGACCTACGAC | GGCCTCCTCCATTAAACCTC | CA352127.1 | 201 |
| **gpx1a** | AATGTGGCGTCACTCTGAGG | CAATTCTCCTGATGGCCAAA | HE687021 | 131 |
| **gpx1b1** | CGAGCTCCATGAACGGTACG | TGCTTCCCGTTCACATCCAC | CA357669.1 | 183 |
| **gpx1b2** | TCGGACATCAGGAGAACTGC | TCCTTCCCATTCACATCCAC | HE687023 | 121 |
| **cat** | TGATGTCACACAGGTGCGTA | GTGGGCTCAGTGTTGTTGAG | BX087110.3 | 195 |
| **gr** | CTAAGCGCAGCGTCATAGTG | ACACCCCTGTCTGACGACAT | CA368976.1 | 108 |
| **gst** | TCGCTGACTGGACGAAAGGA | CGAAGGTCCTCAACGCCATC | BX302932.3 | 196 |
| **b-actin** | GCCGGCCGCGACCTCACAGACTAC | CGGCCGTGGTGGTGAAGCTGTAAC | AF157514 | 73 |
| **elf1a** | TTAAGCAACCATGGGAAAGG | TACCTGCCGGTCTCAAACTT | NM_001124339 | 264 |

Abbreviations: sod: superoxide dismutase; gpx: glutathione peroxidase; cat: catalase, gr: glutathione reductase; gst: glutathione S-transferase; b-actin: beta-actin; elf: elongation factor.

**Table S2.** Correlations between astaxanthin concentrations and MDA and GSH concentrations and antioxidant enzyme activities in the muscle and liver of rainbow trout

| | Diets[1] | | MDA[3] | GSH | GST | GPX | GR | SOD |
|---|---|---|---|---|---|---|---|---|
| Muscle | PM SA | AST[2] | R -0.29 | R 0.25 | R 0.042 | R -0.053 | R 0.46 | R 0.39 |
| | | | P 0.45 | P 0.52 | P 0.92 | P 0.89 | P 0.22 | P 0.30 |
| | PM AA | AST | R 0.46 | R -0.55 | R 0.87 | R 0.51 | R 0.76 | R 0.74 |
| | | | P 0.21 | P 0.13 | P 0.002** | P 0.17 | P 0.018* | P 0.023* |
| | FM SA | AST | R 0.28 | R 0.70 | R 0.35 | R -0.39 | R 0.45 | R 0.21 |
| | | | P 0.46 | P 0.035* | P 0.36 | P 0.30 | P 0.23 | P 0.59 |
| | FM AA | AST | R 0.096 | R 0.44 | R 0.64 | R 0.241 | R 0.059 | R -0.57 |
| | | | P 0.81 | P 0.24 | P 0.066 | P 0.53 | P 0.88 | P 0.11 |
| Liver | PM SA | AST | R -0.83 | R -0.40 | R 0.39 | R 0.14 | R -0.11 | R 0.818 |
| | | | P 0.005** | P 0.29 | P 0.30 | P 0.73 | P 0.78 | P 0.007** |
| | PM AA | AST | R -0.40 | R 0.062 | R 0.72 | R 0.66 | R -0.20 | R 0.65 |
| | | | P 0.29 | P 0.87 | P 0.030* | P 0.055 | P 0.61 | P 0.057 |
| | FM SA | AST | R -0.11 | R -0.58 | R -0.24 | R 0.26 | R 0.30 | R 0.44 |
| | | | P 0.78 | P 0.11 | P 0.54 | P 0.49 | P 0.43 | P 0.24 |
| | | AST | R 0.12 | R 0.27 | R 0.031 | R 0.67 | R 0.37 | R 0.90 |

| | | | | | | | | |
|---|---|---|---|---|---|---|---|---|
| | FM<br>AA | | P 0.77 | P 0.48 | P 0.94 | P 0.048* | P 0.33 | P 0.001** |

[1]PM: plant protein meal based diets, FM: fishmeal based diets; SA: synthetic astaxanthin, AA: microalgal astaxanthin.

[2]AST: tissue concentrations of astaxanthin.

[3]MDA: malondialdehyde; GSH: glutathione; GST: Glutathione S-transferase; GPX: Glutathione peroxidase; GR: Glutathione reductase; SOD: Superoxidase dismutase.

*, **Positive or negative R and $P < 0.05$ represents a positive or negative correlation between the concentration of AST and concentrations of MDA or GSH or activities of GST, GPX, GR, and SOD in a given tissue. *$P < 0.05$; ** $P < 0.01$.

**Table S3.** Stepwise regression analysis of tissue redox biomarkers as dependent variables and dietary AST and DHA concentrations as independent variables in rainbow trout

| Dependent variables (Y)[1] | Significant independent variables (X)[2] | Equations |
|---|---|---|
| | Plant meal-based diet | |
| **Muscle** | **Dietary AST & DHA concentrations** | |
| M[3]-MDA (μM/g protein) | / | / |
| M-GSH (μM/mg protein) | / | / |
| M-GST (mU/mg protein) | $X_0$: D-DHA (mg/g diet) | $Y=0.55+0.25X_0$ |
| M-GPX (mU/mg protein) | / | / |
| M-GR (mU/mg protein) | / | / |
| M-SOD (mU/mg protein) | $X_0$: D-DHA (mg/g diet) | $Y=10+8.7X_0$ |
| **Liver** | **Dietary AST & DHA concentrations** | |
| L-MDA (μM/g protein) | / | / |
| L-GSH (μM/mg protein) | / | / |
| L-GST (mU/mg protein) | / | / |
| L-GPX (mU/mg protein) | / | / |
| L-GR (mU/mg protein) | / | / |
| L-SOD (mU/mg protein) | $X_0$: D-DHA (mg/g diet) | $Y=79+35X_0$ |
| | Fishmeal-based diet | |
| **Muscle** | **Dietary AST & DHA concentrations** | |

| | | |
|---|---|---|
| M-MDA (μM/g protein) | / | / |
| M-GSH (μM/mg protein) | / | / |
| M-GST (mU/mg protein) | $X_0$: D-DHA (mg/g diet) | $Y=0.71+ 0.76X_0$ |
| M-GPX (mU/mg protein) | / | / |
| M-GR (mU/mg protein) | $X_0$: D-DHA (mg/g diet) | $Y=0.149+ 0.045X_0$ |
| M-SOD (mU/mg protein) | / | / |
| **Liver** | **Dietary AST & DHA concentrations** | |
| L-MDA (μM/g protein) | / | / |
| L-GSH (μM/mg protein) | / | / |
| L-GST (mU/mg protein) | / | / |
| L-GPX (mU/mg protein) | $X_0$: D-DHA (mg/g diet) | $Y=4.4+ 5.5X_0$ |
| L-GR (mU/mg protein) | / | / |
| L-SOD (mU/mg protein) | / | / |

[1]Dependent variables (X, tissue concentrations or activities): MDA: malondialdehyde, GSH: glutathione, GST: glutathione S-transferase, GPX: glutathione peroxidase, GR: glutathione reductase, SOD: superoxide dismutase.

[2]Independent variables (Y, dietary concentrations): AST, astaxanthin; DHA, docosahexaenoic acid (refer to reference 15).

[3]D: diets; L: liver; and M: muscle.

**Table S4.** Stepwise regression analysis of tissue redox biomarkers as dependent variables and tissue AST and DHA concentrations and antioxidant enzyme gene mRNA levels as independent variables in rainbow trout

| Dependent variables (Y)[1] | Significant independent variables (X)[2] | Equations |
|---|---|---|
| | Plant meal-based diet | |
| **Muscle** | **Tissue AST & DHA concentrations and gene expression** | |
| M[3]-MDA (μM/g protein) | $X_0$: L-GR (relative mRNA level) | $Y=2.1-0.008X_0$ |
| M-GSH (μM/mg protein) | / | / |
| M-GST (mU/mg protein) | $X_0$: L-CAT (relative mRNA level) | $Y=0.078+0.005X_0$ |
| M-GPX (mU/mg protein) | / | / |
| M-GR (mU/mg protein) | $X_0$: L-CAT (relative mRNA level) | $Y=0.01+ 0.002X_0$ |
| M-SOD (mU/mg protein) | $X_0$: L-CAT (relative mRNA level) | $Y=-4.7+0.18X_0$ |
| **Liver** | **Tissue AST & DHA concentrations and gene expression** | |
| L-MDA (μM/g protein) | $X_1$: L-GST (relative mRNA level) | $Y=2.5+0.011X_1-0.012X_2$ |
| | $X_2$: L-SOD1 (relative mRNA level) | |
| L-GSH (μM/mg protein) | $X_0$: L-GR (relative mRNA level) | $Y=53+0.19X_0$ |
| L-GST (mU/mg protein) | $X_0$: L-SOD2 (relative mRNA level) | $Y=-1.6+0.051X_0$ |
| L-GPX (mU/mg protein) | $X_0$: L-GPX1b2 (relative mRNA level) | $Y=21+0.06X_0$ |
| L-GR (mU/mg protein) | $X_0$: L-GST (relative mRNA level) | $Y=1.2+0.006X_0$ |
| L-SOD (mU/mg protein) | $X_0$: L-DHA (mg/g tissue) | $Y=17+401X_0$ |
| | Fishmeal-based diet | |

| | | |
|---|---|---|
| **Muscle** | **Tissue AST & DHA concentrations and gene expression** | |
| M-MDA (μM/g protein) | / | / |
| M-GSH (μM/mg protein) | $X_0$: L-GPX1a (relative mRNA level) | $Y=210-0.67X_0$ |
| M-GST (mU/mg protein) | $X_1$: L-GPX1a (relative mRNA level) | $Y=1.1-0.004X_1+0.004X_2$ |
| | $X_2$: L- GPX1b1 (relative mRNA level) | |
| M-GPX (mU/mg protein) | / | / |
| M-GR (mU/mg protein) | $X_0$: L-GPX1a (relative mRNA level) | $Y=1.3-0.001X_0$ |
| M-SOD (mU/mg protein) | $X_0$: L-SOD2 (relative mRNA level) | $Y=52-0.221X_0$ |
| **Liver** | **Tissue AST & DHA and gene expression** | |
| L-MDA (μM/g protein) | / | / |
| L-GSH (μM/mg protein) | $X_0$: L-AST (μg/g tissue) | $Y=71+64X_0$ |
| L-GST (mU/mg protein) | $X_1$: L-SOD1 (relative mRNA level) | $Y=14-0.095X_1+0.016X_2$ |
| | $X_2$: L- GR (relative mRNA level) | |
| L-GPX (mU/mg protein) | $X_0$: L-GPX1a (relative mRNA level) | $Y=29-0.13X_0$ |
| L-GR (mU/mg protein) | $X_1$: L-DHA (mg/g tissue) | $Y=3.7+1.4X_1-0.032X_2$ |
| | $X_2$: L-SOD1 (relative mRNA level) | |
| L-SOD (mU/mg protein) | $X_0$: L-CAT (relative mRNA level) | $Y=-73+1.2X_0$ |

[1]Dependent variables (X, tissue concentrations or activities): MDA: malondialdehyde, GSH: glutathione, GST: glutathione S-transferase, GPX: glutathione peroxidase, GR: glutathione reductase, SOD: superoxide dismutase.

[2]Independent variables (Y): tissue concentrations of AST (astaxanthin) and DHA (docosahexaenoic acid, refer to reference 15) and mRNA levels of 8 antioxidant enzyme genes including catalase (CAT) gene.

[3]L: liver, M: muscle.
